# Make the Quality Control of Essential Oils Greener: Fast Enantioselective GC-MS Analysis of Sweet and Bitter Orange as a Case Study

**DOI:** 10.3390/molecules28176231

**Published:** 2023-08-24

**Authors:** Gaia Bechis, Manuel A. Minteguiaga, Barbara Sgorbini, Arianna Marengo, Patrizia Rubiolo, Cecilia Cagliero

**Affiliations:** 1 Department of Drug Science and Technology, University of Turin, 10125 Turin, Italy; gaia.bechis@unito.it (G.B.); mminte@fq.edu.uy (M.A.M.); barbara.sgorbini@unito.it (B.S.); arianna.marengo@unito.it (A.M.); patrizia.rubiolo@unito.it (P.R.); 2 Espacio de Ciencia y Tecnología Química (ECTQ), CENUR Noreste-Tacuarembó, Universidad de la República, Tacuarembó 45000, Uruguay; 3 Laboratorio de Biotecnología de Aromas (LaBiotA), Facultad de Química, Universidad de la República, Montevideo 11800, Uruguay

**Keywords:** essential oils, quality control, green gas chromatography, sweet and bitter oranges

## Abstract

Quality control of essential oils is fundamental for verifying their authenticity and conformity with quality standards, ensuring their safety and regulatory compliance, and monitoring their consistency. Companies that produce or market essential oils routinely evaluate the quality and authenticity of their products. However, they also must deal with increasing attention to environmental sustainability as well as practical considerations such as productivity, cost, and simplicity of methods. In this study, enantioselective gas chromatography (GC) was adopted to evaluate the quality of sweet and bitter orange essential oils, used as a case study. The analytical conditions were optimized and translated to fast GC to evaluate the impact of this approach on the environmental footprint of the analyses. The greenness of fast GC, compared with conventional GC, was quantitatively evaluated using a dedicated metric tool (AGREE), and important improvements have been calculated. The developed methods were applied to a set of commercial essential oils, and the data about the enantiomeric composition and relative percentage abundance were elaborated through multivariate statistics (principal component analysis). The results showed that fast chiral gas chromatography enables the classification of citrus essential oil samples and can be considered an environmentally friendly and sustainable approach for evaluating their quality.

## 1. Introduction

According to ISO 9235:2021, essential oils (EOs) are “products obtained from a natural raw material of plant origin by steam distillation, by mechanical processes from the epicarp of citrus fruits, or by dry distillation after separation of the aqueous phase—if any—by physical processes” [1]. Their analysis as part of quality control is essential for verifying authenticity, meeting quality standards, ensuring safety and regulatory compliance, monitoring consistency, and supporting research and development efforts [2].

These controls are very important, considering that the global market for EOs is estimated at more than $8 billion and is continuously growing [3,4]. Within the EOs market, the citrus segment has a dominant share of about 40%. EOs from citrus fruits are typically obtained by industrial cold pressing extraction and used in a variety of industrial fields, from food and beverages to cosmetics (with extensive use in fragrances) and pharmaceuticals [5,6,7]. In the food sector, they are mainly used as flavors since many citrus EOs and products made from them (e.g., concentrated EOs, terpeneless, etc.) are generally recognized as safe (GRAS) by the Expert Panel of the Flavor and Extract Manufacturers Association (FEMA) [8]. Some of them can also be used as botanicals in dietary supplements. The EOs of citrus fruits are composed of a volatile and a non-volatile fraction and are extracted mainly from the fruit peels by cold pressing. The volatile components, which account for between 85% and 99% of the total oil, consist mainly of monoterpene hydrocarbons, followed by sesquiterpene hydrocarbons, their oxygenated derivatives, and aliphatic aldehydes, alcohols, esters, and ketones [5]. The non-volatile residue, which represents between 1% and 15% of the oil, can contain hydrocarbons, sterols, fatty acids, waxes, carotenoids, coumarins, psoralens, and flavonoids [5]. The chemical characterization of the composition of citrus oils is described exhaustively by Dugo and Mondello [5]. Among the EOs from citrus, the most produced (and also consumed as a flavoring agent) is certainly the sweet orange oil extracted from the fruit peels of *Citrus × aurantium* L. (ex *Citrus sinensis* (L.) Osbeck), while, in contrast, the sour/bitter orange essential oil (ex *Citrus aurantium* L.) is characterized by lower production [8] and consequently higher economic value. Both sweet oranges (SO) and (sour/)bitter oranges (BO) are currently included under the same taxon, *Citrus × aurantium* L., highlighting their hybrid character [9]. However, the taxonomy of citrus species is currently a matter of debate in the literature. Despite a very similar composition [5], SO and BO oils have different organoleptic properties that distinguish them from each other in terms of aroma [10].

As mentioned earlier, companies that produce or commercialize orange EOs need to evaluate their composition in order to make an accurate judgment of quality and authenticity. At the same time, they must deal with increasing regulatory and public attention to environmental sustainability, as well as considerations such as productivity, costs, and simplicity of methods along the entire value chain [11]. To improve the environmental footprint of these controls, analysts are advised to follow the twelve principles of Green Analytical Chemistry (GAC) [12]. Due to the volatility of most of the components, quality control of orange EOs is usually performed by gas chromatography (GC) [6]. According to the GAC principle, several strategies can be used to increase the greenness of these analyses. The first is to improve the amount of information obtained with a single analysis (Principle 8). Enantioselective GC can be very useful in this regard, providing qualitative and semi-quantitative information on both chiral and non-chiral compounds. In addition, enantiomeric recognition can make a fundamental contribution to (1) define the biosynthetic pathway of a compound; (2) correlate the chemical structures to the organoleptic properties; (3) characterize a sample; (4) confirm the geographical origin of a sample; and (5) detect possible adulterants [13,14]. Other strategies to improve the environmental footprint of GC analysis include (i) miniaturization; (ii) replacing helium with hydrogen (from non-fossil sources); and (iii) reducing analysis time [15,16,17]. The latter is beneficial not only in terms of reducing energy and material consumption (carrier gas) but also in terms of improving productivity. Although these strategies are often mentioned as approaches to increasing the greenness of GC analyses, a quantitative assessment of the environmental impact of GC and fast GC analyses has seldom been conducted [18]. In this sense, the use of metric tools to evaluate the greenness of a method is of extreme importance. One of the newest and most widely used tools is the Analytical GREEness Calculator (also called AGREE) [19], which evaluates the compliance of an analytical method with the twelve principles of the GAC and provides an overall score for the method under study on a scale of 0–1 (where 1 is the best score and 0 is the worst score), as well as an overview of the contribution of each criterion to the total impact of the method.

Thus, the aim of this study was to quantitatively evaluate the improvement in the greenness of fast GC compared with conventional GC in the chiral analysis of essential oils. The quality evaluation of EOs from sweet and bitter oranges served as a case study. The analysis of the studied oils was optimized and evaluated in terms of greenness. The developed methods were applied to a set of commercial EOs to determine if the proposed approach is able to discriminate between different samples with the support of multivariate statistical elaboration.

## 2. Results and Discussion

### 2.1. Sweet and Bitter Orange Essential Oil Characterization

As mentioned in the introduction, the essential oils of sweet (SO) and bitter (BO) oranges show a high degree of similarity in terms of chemical constituents. Table S1 (Supplementary Materials) shows the comparison between the percentage composition of the volatile fraction of the two cold-pressed oils reported in the literature and summarized by Dugo and Mondello [5]. Limonene is certainly the main constituent of the oils, with a relative abundance of more than 85% for both, followed by myrcene (which can reach values of up to 2.5–3%), and then by other minor hydrocarbons, aldehydes, alcohols, ketones, and esters. The selection of markers specific to the two oils is a challenging task because their compositions are very similar. Some differences can be seen, for example, in the abundance of δ-3-carene, which is reported in the ranges 0–0.02% and 0.04–0.31% for the BO and SO EOs, respectively, and in the abundance of linalyl acetate (0.07–2.72% in BO and 0–0.1% in SO), but a clear differentiation of the two oils based on percentage composition comparison is almost impossible. Evaluation of the enantiomeric composition of the EOs may be helpful in this regard, as some differences have been reported for the two oils (see Table S1). For example, β-pinene exhibits a high enantiomeric excess for the (*S*)-enantiomer in BO EOs, while the percent enantiomeric composition (EC%) is more variable in SO, where the (*R*)-enantiomer may even be the most abundant. In contrast, sabinene exhibits a higher enantiomeric excess of the (*R*)-enantiomer in SO than in BO. The most striking marker is linalool, which has an opposite enantiomeric distribution in the two oils. Indeed, literature data report an enantiomeric excess of (*R*)-linalool (EC%: 61.1–92.4%) in BO, while the (*S*)-enantiomer is more abundant in SO (EC%: 81.1–97.8%). This difference in composition may also affect the organoleptic properties of the two oils, as it is known that the two enantiomers of the same molecule can differ in odor quality and potency (Table S2). In the case of linalool, for instance, the (*R*)-enantiomer is reported to be “*floral-woody, with lavender note*”, while the (*S*)- is “*fresh, floral, petitgrain-like*” [20].

Based on the above considerations, the simultaneous determination of the relative abundance of the constituents of orange EOs and their enantiomeric distribution can be used to distinguish between different samples and evaluate their quality. As mentioned in the introduction, enantioselective GC, which provides qualitative and semi-quantitative information on chiral and non-chiral compounds, can be considered the technique of choice for this purpose. Among the four cyclodextrin derivatives most commonly used in the essential oil field [14,21], the 2^I-VII^-*O*-ethyl-3^I-VII^-*O*-ethyl-6^I-VII^-*O*-*tert*-butyldimethylsilyl-β-cyclodextrin allows the separation of all orange EO markers and was therefore selected for this study.

Two authentic essential oils of SO and BO were first analyzed by enantioselective GC-MS on a conventional column (25 m length × 0.25 mm *d_c_*, 0.25 µm *d_f_*) under standard conditions usual for chiral GC analyses, i.e., a temperature program of 2 °C min^−1^ and carrier gas flow of 1 mL min^−1^. Under these conditions, it was indeed possible to use the databases of linear retention indices of enantiomers of natural chiral compounds previously developed in the authors’ laboratory [21] to identify the target analytes and also to assign the enantiomeric configurations to the separated chiral compounds.

**Figure 1:**
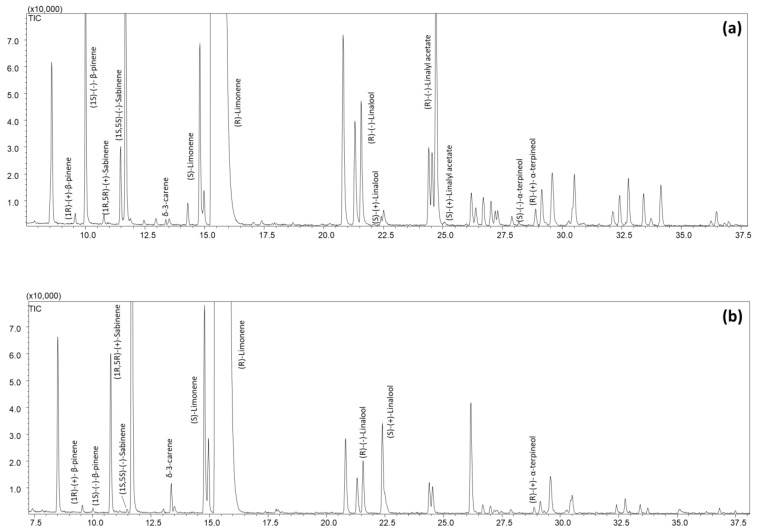
GC-MS profiles of the reference bitter orange (**a**) and sweet orange (**b**) essential oil on the 2^I-VII^-*O*-ethyl-3^I-VII^-*O*-ethyl-6^I-VII^-*O*-*tert*-butyldimethylsilyl-β-cyclodextrin coated column (25 m length × 0.25 mm *d_c_*, 0.25 µm *d_f_*). Temperature program: 50.0 °C to 220 °C (5 min) at 2 °C min^−1^. Only marker compounds are indicated.

Figure 1 shows the comparison of the GC-MS profiles of the reference SO and BO EOs samples.

### 2.2. Improvement in the Enviromental Footprint of the Chiral Analyses of Citrus Essenial Oils

#### 2.2.1. Optimization and Speeding-Up of the Analyses

As mentioned in the introduction, the first approach to improving the greenness of a gas chromatographic analysis is to speed it up. This would be beneficial not only to increase the productivity of the laboratory but also to reduce the consumption of energy and save carrier gas.

**Figure 2:**
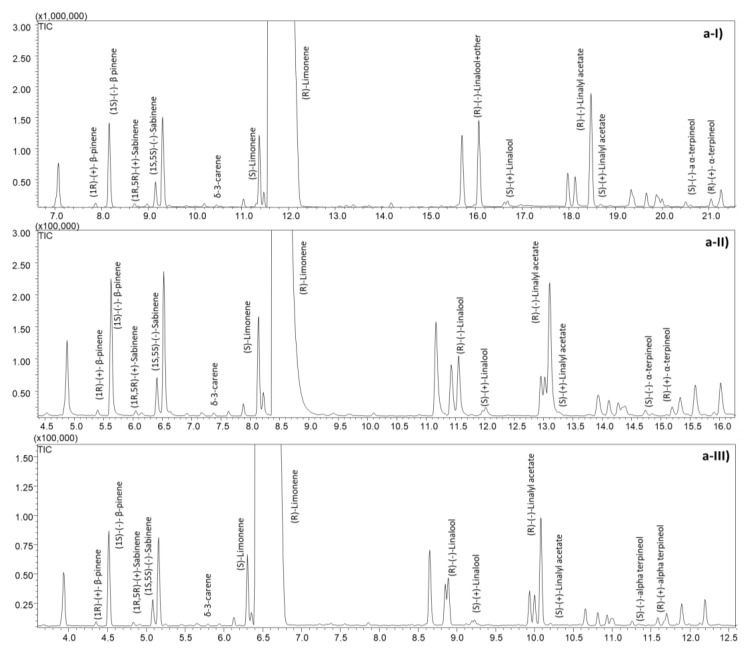

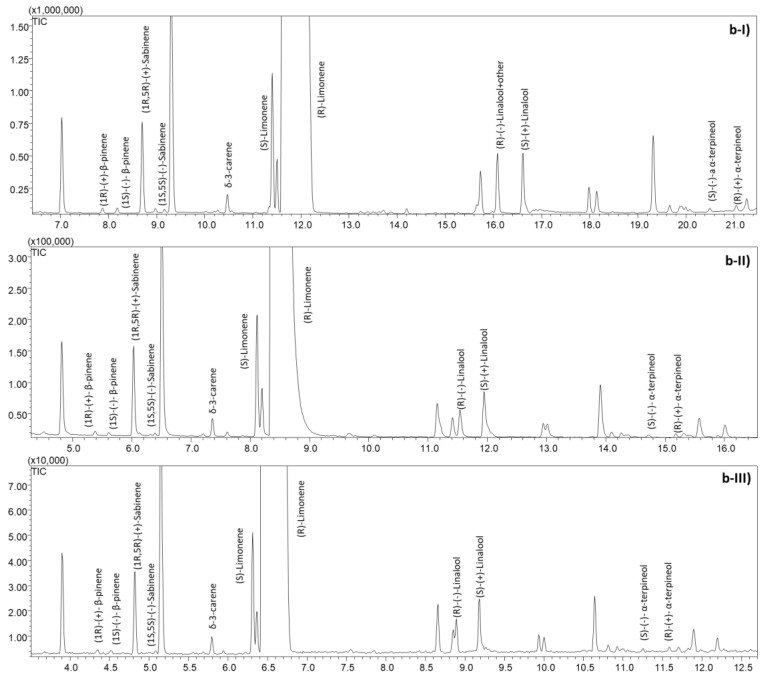
Optimized GC-MS profiles of the reference bitter orange (**a**) and sweet orange (**b**) essential oil on the 2^I-VII^-*O*-ethyl-3^I-VII^-*O*-ethyl-6^I-VII^-*O*-*tert*-butyldimethylsilyl-β-cyclodextrin coated columns (I: 25 m length × 0.25 mm *d_c_*, 0.25 µm *d_f_*, II: 15 m length × 0.18 mm *d_c_*, 0.18 µm *d_f_*, III: 10 m length × 0.10 mm *d_c_*, 0.10 µm *d_f_*). Temperature programs: see Section 3.3.

The speeding-up of enantioselective GC separation was achieved by a strategy [13] that consisted of (i) optimizing the chromatographic conditions that provide the best compromise between speed and separation with a conventional column and (ii) translating the method to columns with reduced geometry. Two higher temperature rates (3 and 5 °C min^−1^) were first tested on the conventional column. Acceptable separation between the enantiomers of the chiral pairs, as well as minimal co-elution between the target compounds (overcome by selecting specific ions for the analyte under study), was observed at 3 °C min^−1^. In contrast, a higher degree of co-elution and, more importantly, a lack of separation between the enantiomers of linalyl acetate occur at a temperature rate of 5 °C min^−1^, making this condition unsuitable for the analysis of the oils under study. In addition, all target analytes were eluted below 120 °C, so the temperature rate was increased to 10 °C min^−1^ afterwards. The optimized multi-ramp temperature program on the conventional column was the following: 50 °C to 120 °C at 3 °C min^−1^ and then increasing to 220 °C at 10 °C min^−1^. It allowed a drastic reduction in analysis time by two-thirds, from 87 min to 31 min (see Figure 1 and Figure 2(a-I,b-I)).

To further speed up the analysis, two columns with the same stationary phase and reduced dimensions were tested: a 15 m × 0.18 mm *d_c_*, 0.18 µm *d_f_* and a 10 m × 0.10 mm *d_c_*, 0.10 µm *d_f_*. The analytical conditions for these two narrow-bore columns were translated from the original method using an appropriate GC method translation software [22], based on the approach proposed by Klee and Blumberg [23]. For the 15 m column, the translated conditions suggest reducing the carrier gas flow to 0.72 mL min^−1^ and increasing the initial temperature rate to 5.5 °C min^−1^. However, adopting the translated temperature conditions, an unresolvable co-elution occurs between the (*R*)-enantiomer of linalool and an interfering compound, and therefore, the temperature rate was decreased to 4 °C min^−1^. The optimized temperature program on the 15 m column (see Section 3.3) allows for to attainment of a chromatographic profile comparable with the conventional column and to separate and quantify all target markers in only 22 min (see Figure 2(a-II,b-II)). Finally, the method developed for this column was translated to the 10 m column. In this case, the flow rate was further reduced to 0.4 mL min^−1^ and the initial temperature program increased to 5.48 °C min^−1^ without observing additional co-elutions (see Figure 2(a-III,b-III)). Although the analysis time with this last column is the lowest (16 min), it is important to emphasize that the use of a 0.10 µm *dc* column in routine quality control may have some limitations. In particular, the high resistance of these columns due to their small diameter requires high inlet pressures (more than 200 kPa), which in some cases require the use of dedicated electronic pressure controls. In addition, these columns are characterized by a lower loading capacity, which requires a large increase in the split ratio (100:1 in this study). This limit is particularly constraining when, as in this application, one component is very abundant and its overload needs to be reduced without decreasing the amount of the other minor EO components below the detection limit.

The enantiomeric composition of the two reference EOs on the three columns studied was then determined and compared to evaluate the accuracy of all methods developed. Table S3 (Supplementary Materials) reports the results of the EC% and shows that the measured enantiomeric distribution is equivalent despite the column used.

#### 2.2.2. Assessment of the Environmental Footprint of the Methods

The improvement in the environmental footprint of the methods optimized for the three investigated columns was then evaluated by comparing, through the AGREE metric, their “greenness” performance against the standard method.

**Figure 3:**
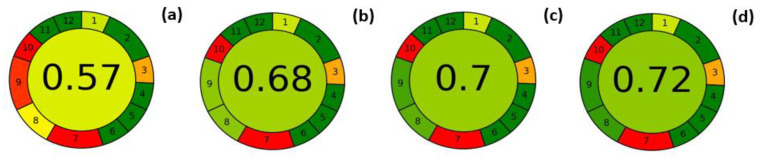
AGREE scores calculated for the standard method for the enantioselective GC-MS analysis of chiral markers in BO and SO (**a**), and that for the corresponding optimized methods developed with the 25 m (**b**), 15 m (**c**) and 10 m (**d**) columns.

Figure 3 shows the pictograms of the comparisons obtained for the standard method (a) and the proposed methods (b–d), while Figures S1–S4 of the Supplementary Material show the detailed reports of each evaluation.

The standard method shows a final score of 0.57 and several criteria with a score of less than 0.5, suggesting that these aspects should be improved to meet the 12 principles of GAC. Criticisms of the criteria related to direct analyses (Principles 1 and 3) cannot be eliminated because essential oils must be analyzed with a proper dilution and sample preparation, even if minimal, cannot therefore be avoided. The low score of Principle 7 (generation of waste) is related to the high consumption of carrier gas (about 2 L per analysis); this value can be reduced by decreasing the analysis time and/or the flow rate. Criterion 8 takes into account the number of analytes determined in a single run (quite good) and sample throughput, the latter of which can be significantly improved. Another critical point of the standard method in terms of greenness is the energy consumption (principle 9), which was measured at 1.4 kWh. Finally, the use of non-renewable materials (cyclohexane and helium) drops the score for principle 10 to the lowest value. This parameter can be improved by using a renewable solvent (e.g., ethanol) to dilute the samples, but this could damage the cyclodextrin-based stationary phase due to some water content in the alcoholic solvent. Regarding the use of alternative carrier gas to fossil fuel-derived gases, it is noted that manufacturers are moving towards replacing helium with hydrogen (possibly from non-fossil sources), including for GC-MS, but this requires the use of dedicated instruments.

The green performance of the optimized methods increases dramatically. A value of 0.68 out of 1 is obtained for the optimized method on the conventional 25 m column; this value further increases to 0.7 and 0.72 for the optimized methods on the 15 m and 10 m columns, respectively. This improvement is mainly due to the better performance associated with principles 8 and 9. Indeed, the sample throughput increases from 0.7 analyses per hour for the reference method to 1.9, 2.7, and 3.8 analyses/hour for the optimized methods on the 25 m, 15 m, and 10 m columns, respectively. Furthermore, the energy consumption shows a drastic reduction: the value of 1.4 kWh/analysis for the standard method decreases to 0.5 kWh, 0.3 kWh, and 0.2 kWh per analysis for the optimized methods on the 25 m, 15 m, and 10 m columns, respectively. Surprisingly, the value for waste (Principle 7) does not improve, although helium consumption decreases from 2 L for the standard method to 0.7 L, 0.4 L, and 0.2 L for the optimized methods. The same low score is due to the fact that waste volumes greater than 200 mL are considered highly polluting. This reference value can be considered reasonable when it relates to sample preparation and, more generally, to liquids. However, it can be considered a limitation when comparing the performance of GC analyses, where carrier gas consumption is inevitably higher but differs greatly when modulating the chromatographic conditions.

Based on the above considerations, it should be noted that the optimized conditions on the 15 m × 0.18 mm *d_c_*, 0.18 µm *d_f_* column allow for reliable data and optimal performance in terms of greenness while maintaining good column capacity and method simplicity. Therefore, this method can be considered the optimal choice for a quality assessment of SO and BO EOs and was therefore selected for further determination.

### 2.3. Analysis of Commercial Essential Oil Samples

#### 2.3.1. Stability of the Enantiomeric Composition over Time

Besides the similar chemical composition of SO and BO, another challenge in the classification of these EOs is the possible change in their composition due to aging and/or imperfect storage, which could lead to the very well-known oxidation of limonene [24]. Therefore, the stability of the enantiomeric distribution over time was studied to determine if it could be considered a characteristic of the sample regardless of age and storage conditions. In this study, the enantiomeric composition of the chiral marker linalool of two samples from the authors’ laboratory, BO and SO, respectively, prepared and first analyzed in 2006 and stored at room temperature, was investigated. The chromatographic profiles of the two oils highlight a strong degradation of limonene, but as can be seen in Table 1, the enantiomeric composition of linalool is perfectly preserved. The data are also confirmed by the analyses of a sample of linalool isolated from bergamot essential oil in 2016, which also retained its enantiomeric composition over time.

#### 2.3.2. Multivariate Analysis of the Distribution of the Target Compounds in Commercial Samples

A series of 29 commercial samples of BO and 19 commercial samples of SO EOs were then analyzed with the optimized method. The enantiomeric composition of the chiral markers β-pinene, sabinene, limonene, linalool, linalyl acetate, and α-terpineol was determined, as was there area percentage in the oil. The percentage of δ-3-carene was also evaluated because, as reported in Section 2.1, it can help to distinguish between the two oils studied.

**Figure 4:**
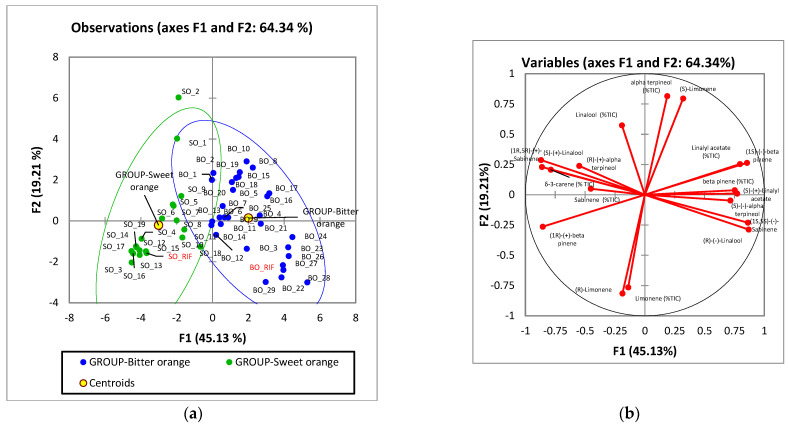
Principal component analysis relative to the distribution of orange essential oils based on the % abundance and enantiomeric distribution. (**a**) scores plot and (**b**) loadings plot. Legend: green: sweet orange (SO), blue: bitter orange (BO). SO_RIF: reference of an authentic SO EO, BO_RIF: reference of an authentic BO EO.

The results (reported in Table S4) were elaborated by unsupervised multivariate statistical analysis. First, a principal component analysis (Figure 4) was performed to investigate the natural clustering between samples. The results show that the composition of most SO and BO EOs is similar to that of the reference, but some others differ and the two clusters partially overlap (Figure 4a). The main differences between SO and BO EOs are highlighted in the first component (PC1, which explains 45.13% of the variability). The loading plot (Figure 4b) shows the distribution of variables. As expected, a higher percentage of (*R*)-linalool, (*S*)-β-pinene, and total linalyl acetate are positively correlated with BO, and this EO also shows a higher relative percentage amount of β-pinene and a higher percentage of (−)-sabinene. In contrast, SO generally shows higher amounts of (S)-linalool, (*R*)-β-pinene, and δ-3-carene, as well as higher amounts of total sabinene and its (+)-enantiomer. The PC2 (which explains 19.21% of the variability) seems instead to correlate more with the conservation status of the EO, in particular with the total percentage of limonene, which is lower than the literature range for BOs 8 and 10 and SOs 1 and 2.

**Figure 5:**
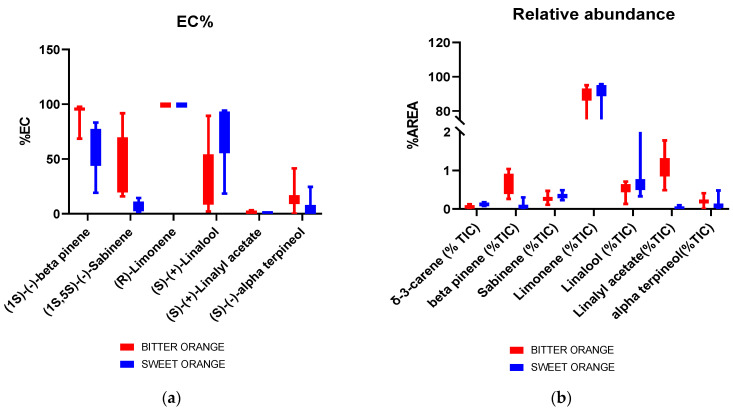
Boxplots relative to the enantiomeric composition % (**a**) and % relative abundance (**b**) for BO and SO. For the enantiomeric composition, the EC% of only one enantiomer is reported to avoid redundancies.

The different distribution of the markers in SO and BO EOs is also highlighted in the boxplots reported in Figure 5. Indeed, a generally different enantiomeric excess of β-pinene, sabinene, and linalool (Figure 5a) can be seen, as well as a different relative abundance of δ-3-carene, β-pinene, and linalyl acetate (Figure 5b).

## 3. Materials and Methods

### 3.1. Samples and Chemicals

Cyclohexane and a standard mixture of *n*-Alkanes (from *n*-7 to *n*-30 at a concentration of 1 mg mL^−1^) were obtained from Merck (Milan, Italy). Cyclohexane was used as a dilution solvent for the essential oils and linalool and linalyl acetate samples. 

Two authentic bitter (BO) and sweet (SO) orange (*Citrus × aurantium* L. [9]) essential oils and linalool isolated from bergamot essential oil were kindly provided by an Italian local producer. A set of 29 bitter orange and 19 sweet orange EOs was purchased in local shops. One BO and one SO essential oil produced in 2006 from the authors’ laboratory collection were also analyzed. The EO samples were diluted at a concentration of 6 mg mL^−1^ in cyclohexane before analysis, while linalool and linalyl acetate were diluted at a concentration of 0.1 mg mL^−1^ in the same solvent. At the same time, the alkane standard mixture was diluted in cyclohexane at a concentration of 0.1 mg mL^−1^ and applied for linear retention indices (*I^T^_s_*) calculation.

### 3.2. Instruments

GC-MS analyses were carried out on a Shimadzu GC-MS system consisting of a Shimadzu GC2010 gas chromatograph coupled with a Shimadzu QP2010 Plus mass spectrometer (Shimadzu, Milan, Italy). A MultiPurpose Sampler MPS2 (Gerstel, Mülheim a/d Ruhr, Germany) was adopted as an autosampler.

The energy consumption was measured with a Zhurui PR10 power meter plug (Zhurui, China).

### 3.3. Analysis Conditions

Separation of target compounds was achieved with the MEGA-DEX DET-Beta capillary column coated with 30% 2^I-VII^-*O*-ethyl-3^I-VII^-*O*-ethyl-6^I-VII^-*O*-*tert*-butyldimethylsilyl-β-cyclodextrin diluted in PS086 from MEGA (MEGA S.r.l., Legnano, MI, Italy). 

Different column dimensions were tested: (a) 25 m length × 0.25 mm *d_c_*, 0.25 µm *d_f_*, (b) 15 m × 0.18 mm *d_c_*, 0.18 µm *d_f_*, (c) 10 m × 0.10 mm *d_c_*, 0.10 µm *d_f_*. The analysis conditions for column (a) were as follows: injection temperature: 220 °C, carrier gas: helium, flow: 1.0 mL min^−1^ (pressure 39.9 kPa), injection mode: split, split ratio: 20:1, injection volume: 1 µL. The oven temperature program was set from 50.0 °C to 220 °C (2 min) at 2 °C min^−1^ (total analysis time: 87 min) for the conventional method and from 50.0 °C to 120 °C at 3 °C min^−1^ and then to 220 °C at 10 °C min^−1^ (total analysis time: 31 min) for the optimized method. The analysis conditions for column (b) were: as follows injection temperature: 220 °C, carrier gas: He, flow: 0.72 mL min^−1^ (pressure: 77.7 kPa), injection mode: split, split ratio: 50:1, injection volume: 1 µL. The oven temperature program was as follows: from 50 °C to 120 °C at 4 °C min^−1^ and then to 220 °C at 18 °C min^−1^ (total analysis time: 22 min). Analysis conditions for column (c) were as follows: injection temperature: 220 °C, carrier gas: He, flow: 0.4 mL min^−1^ (pressure: 250.8 kPa), injection mode: split, split ratio: 100:1, injection volume: 1 µL. The oven temperature program was as follows: from 50 °C to 120 °C at 5.48 °C min^−1^ and then to 220 at 25 °C min^−1^ (total analysis time: 16 min).

For all analyses, the MS operative conditions were as follows: transfer line: 230 °C, ion source temperature: 200 °C. The MS operated in electron ionization mode (EI) at 70 eV with a scan rate of 666 amu/s and a mass range of 35–350 m/z.

A summary of all GC analysis conditions is reported in Table S5.

The identification was carried out by a spectral similarity match estimated over commercial and in-house databases. In addition, the enantiomer stereochemistry was confirmed through authentic enantiomeric standards and *I*^T^ comparison with an in-house database of retention indexes with a tolerance of ±3 [21]. The enantiomeric distribution was calculated in terms of percent enantiomeric composition (*EC*%) on specific target ions for each chiral pair (see Table S2) using the following equation:

$$EC\%=\frac{E_{1\;(or\;2)}}{E_{1}+E_{2}}\times 100$$

where *E*_1_ and *E*_2_ are the areas of the first and second eluting enantiomers, respectively.

### 3.4. Data Processing

The software used for data acquisition and processing was GCMSsolution^®^ 4.30 (Shimadzu, Milan, Italy).

The Analytical GREENess Calculator (AGREE v0.4 2020) [19] was applied for the calculation of the greenness score of the methods.

Principal component analysis was carried out with the XLSTAT statistical and data analysis solution (Addinsoft 2020, New York, NY, USA). A heatmap was created using Morpheus software (https://software.broadinstitute.org/morpheus, accessed on 30 June 2023) and Box-Plots by GraphPad Prism 9.3.0.463 (GraphPad Software, LLC, San Diego, CA, USA). Excel software (Microsoft Office v. 2016) was employed for the remaining calculations.

## 4. Conclusions

The results reported in this study show that fast chiral gas chromatography can be considered an environmentally friendly and sustainable approach to evaluating the quality of citrus essential oils, which can be routinely applied in quality control laboratories. In fact, the enantiomeric distribution is stable over time and can be useful in distinguishing different samples when the simple assessment of the relative composition of the chemical components of the essential oils under study has some limitations. The optimization of the chromatographic conditions and the reduction in the column dimensions allow a drastic reduction in the analysis time, with improvements not only in terms of productivity but also in terms of environmental impact since both energy and carrier gas consumption are significantly reduced.

The development of specific and user-friendly metric tools to assess the greenness of an analytical method, such as AGREE, allows laboratories to evaluate and compare the environmental footprint of their methods. However, further improvements are expected to more accurately quantify the contribution of all elements associated with gas chromatographic analysis and to align considerations of environmental friendliness of analyses with considerations of analytical performance and practicality in the context of quality control.

## Figures and Tables

**Table 1:** Mean enantiomeric distribution (EC%) of linalool in different samples over time. RSD% of replicate analyses (*n* = 3) < 5%.

Sample	Year of Production	Original EC%	EC% in 2023
Bitter orange essential oil	2006	(*R*)-Linalool 72.3% (*S*)-Linalool 27.7%	(*R*)-Linalool 72.7% (*S*)-Linalool 27.3%
Sweet orange essential oil	2006	(*R*)-Linalool 7.7% (*S*)-Linalool 92.3%	(*R*)-Linalool 7.8% (*S*)-Linalool 92.2%
Linalool standard (isolated)	2016	(*R*)-Linalool 96.1% (*S*)-Linalool 3.9%	(*R*)-Linalool 95.7% (*S*)-Linalool 4.3%

## Data Availability

The data presented in this study are available on request from the corresponding authors.

## Supplementary Materials

The following supporting information can be downloaded at: https://www.mdpi.com/article/10.3390/molecules28176231/s1, Table S1: percentage composition and enantiomeric distribution of the volatile fraction of the cold-pressed essential oils of sweet (SO) and bitter orange (BO) reported in literature and summarized by Dugo and Mondello; Table S2: odor threshold and odor notes of the enantiomers of the investigated compounds; Table S3: percentage enantiomeric composition of the reference sweet and bitter orange EOs obtained with optimized methods on the three investigated columns; Table S4: percentage enantiomeric composition and percentage relative composition + standard deviation (*n* = 3) of the target compounds for the reference commercial EOs samples; Table S5: Summary of the conventional and optimized GC analyses conditions adopted in this study; Figure S1: Pictograms and reports obtained with AGREE for the conventional method on the 25 m column; Figure S2: pictograms and reports obtained with AGREE and for the optimized method on the 25 m column; Figure S3: pictograms and reports obtained with AGREE and for the optimized method on the 15 m column; Figure S4: pictograms and reports obtained with AGREE for the optimized method on the and 10 m column.

## Sample Availability

Samples of essential oils are available from the authors.
