# Supplementary material for: Make the Quality Control of Essential Oils Greener: Fast Enantioselective GC-MS Analysis of Sweet and Bitter Orange as a Case Study
Source: Molecules. 2023 Aug 24;28(17):6231. doi: 10.3390/molecules28176231 (PMC10488983; doi:10.3390/molecules28176231)
Supplement: Supplementary file 1 [file molecules-28-06231-s001.zip › molecules-2522220-supplementary.pdf]

Supplementary Material

Make the quality control of essential oils greener. Fast enantioselective GC-MS analysis of sweet and bitter orange as a case study

Gaia Bechis [1], Manuel A. Minteguiaga [1,2], Barbara Sgorbini [1], Arianna Marengo [1], Patrizia Rubiolo [1] and Cecilia Cagliero [1,*]

1 Department of Drug Science and Technology, University of Turin, 10125 Turin, Italy; gaia.bechis@unito.it (G.B.), barbara.sgorbini@unito.it (B.S.); arianna.marengo@unito.it (A.M.); patrizia.rubiolo@unito.it (P.R.)
2 Laboratorio de Biotecnología de Aromas, Departamento de Química Orgánica, Facultad de Química, Universidad de la República; m.minte@fq.edu.uy
* Correspondence: cecilia.cagliero@unito.it

Table of contents

Table S1: percentage composition and enantiomeric distribution of the volatile fraction of the cold-pressed essential oils of sweet (SO) and bitter orange (BO) reported in literature and summarized by Dugo and Mondello; Table S2: odor threshold and odor notes of the enantiomers of the investigated compounds; Table S3: percentage enantiomeric composition of the reference sweet and bitter orange EOs obtained with optimized methods on the three investigated columns; Table S4: percentage enantiomeric composition and percentage relative composition + standard deviation (n = 3) of the target compounds for the reference commercial EOs samples; Table S5: Summary of the conventional and optimized GC analyses conditions adopted in this study; Figure S1: Pictograms and reports obtained with AGREE for the conventional method on the 25 m column; Figure S2: pictograms and reports obtained with AGREE and for the optimized method on the 25 m column; Figure S3: pictograms and reports obtained with AGREE and for the optimized method on the 15 m column; Figure S4: ictograms and reports obtained with AGREE for the optimized method on the and 10 m column.

Table S1: Percentage composition and enantiomeric distribution of the volatile fraction of the cold-pressed essential oils of sweet and bitter orange reported in literature and summarized by Dugo and Mondello [1].

	Composition (%)		*% Enantiomeric composition (R)-enantiomer*	
	**Bitter orange**	**Sweet orange**	**Bitter orange**	**Sweet orange**
		Hydrocarbons		
<u>*Monoterpenes*</u>				
δ-3-Carene	0-0.02	0.04-0.31		
p-Cymene	0-0.5	0-0.2		
Limonene	86-95.5	86.18-96.80	99.3-99.5	98.9-100
Myrcene	0.04-3.1	0.93-2.49		
(E)-β-Ocimene	0.01-0.75	0-0.1		
α-Phellandrene	0-0.13	0-0.18	74.9	n.r.
β-Phellandrene	0-0.5	0-0.3	0.56-5.7	0.4-1.4
α-Pinene	0.21-1.2	0.28-1.4	79.7-97.4	90.1-99.7
β-Pinene	0.09-1.6	0-0.24	1.3-7.9	10.6-70.2
Sabinene	0.07-0.45	0.13-0.93	42.1-80.6	94.6-98.8
α-Terpinene	0-0.22	0-1.7		
γ-Terpinene	0-0.73	0-0.33		
Terpinolene	0-0.72	0-0.08		
α-Thujene	0-0.22	0-0.03	69.2-89.7	38-89.9
<u>*Sesquiterpenes*</u>				
Bicyclogermacrene	0-0.28	0-0.01		
β-Bisabolene	0-0.29	0-0.02		
β-Caryophyllene	0.02-0.27	0-0.06		
β-Elemene	0-0.09	0-0.03		
Germacrene D	0.08-0.14	0-0.02		
Valencene	0-0.47	0-0.38		
		Aldehydes		
<u>*Aliphatics*</u>				
Decanal	0.09-0.22	0.12-0.72		
(E)-2-Decenal	0.01-0.15	tr		
Dodecanal	*0-0.04*	0-0.11		
Nonanal	0-0.11	0.02-0.11		
Octanal	0.04-0.24	0.04-0.41		
<u>*Monoterpenes*</u>				
Citronellal	*0-0.07*	0-0.15	57.5	31.3-87.8
Geranial	0.01-0.16	0.04-0.3		
Neral	0-0.1	0.02-0.2		
Perilla aldehyde	0-0.53	0-0.06		
		Ketones		
<u>*Sesquiterpenes*</u>				
Nootkatone	0-0.39	0-0.03		
		Alcohols		
<u>*Aliphatics*</u>				
Octanol	0-0.33	0-0.24		

<u>*Monoterpenes*</u>				
cis-Carveol	0-0.05	0-0.5		
trans-Carveol	0-0.15	0-0.3		
Geraniol	0-0.3	0-0.09		
Linalol	0.1-3.24	0.12-2.56	61.1-92.4	2.2-17.9
Nerol	0-0.17	0-0.05		
Terpinen-4-ol	0-0.4	0-0.31	28.5-34.7	n.r.
α-Terpineol	0.01-2.94	0.02-0.25	70.2-93.2	84.3-94.9
*Thymol**	0-0.12	n.d.		
<u>*Sesquiterpenes*</u>				
(E)-Nerolidol	0.02-0.23	tr		
		Esters		
<u>*Aliphatics*</u>				
Octyl acetate	0.01-0.11	0-0.03		
<u>*Monoterpenes*</u>				
Geranyl acetate	0-0.38	0-0.04		
Linalyl acetate	0.07-2.72	0-0.1	99.2-99.4	n.r.
Neryl acetate	0.01-0.11	0-0.06		
α-Terpinyl acetate	0-0.08	0-0.01		
		Ethers		
<u>*Monoterpenes*</u>				
cis-Limonene oxide	0-0.05	0-0.08		
trans-Limonene oxide	0-0.1	0-0.05		
cis-Linalol oxide	0-0.15	tr		
trans-Linalol oxide	0-0.29	tr		

n.r.: not reported; n.d.: not detected, tr: traces. (*) Thymol is a phenol, but it is included with alcohols for simplicity.

[1] Dugo, G.; Mondello, L. Citrus oils: composition, advanced analytical techniques, contaminants, and biological activity; CRC press: 2010.

Table S2: Odor threshold and odor notes of the enantiomers of the investigated compounds. n.a.: not available

COMPOUND	ODOR THRESHOLD	ODOR NOTE
(1R)-(+)-β-Pinene	2.54 ppm[a]	Sweet fresh pine woody hay green
(1S)-(-)- β-Pinene	4.16 ppm[a]	Characteristic aroma of gum turpentine with a dry, woody or piney, resinous odor of low tenacity[b]
(1R,5R)-(+)-Sabinene	n.a.	n.a.
(1S,5S)-(-)-Sabinene	n.a.	n.a.
(S)-Limonene	0.2 ppm[c]	Fresh, natural, citrusy, orange-like[d]
(R)-Limonene	0.01 ppm[c]	Harsh, turpetine-like, lemon-note[d]
(R)-(-)-Linalool	0.0008 ppm[c]	Floreal-woody, lavender note[e]
(S)-(+)-Linalool	0.0074 ppm[c]	Fresh, floral, petitgrain-like[e]
(R)-(-)-Linalyl acetate	n.a.	n.a.
(S)-(+)-Linalyl acetate	n.a.	n.a.
(S)-(-)-α-Terpineol	9180 ppm[a]	Coniferus, tarry, cold pipe-like[f]
(R)-(+)- α-Terpineol	6800 ppm[a]	Heavy, floral, lilac-like[f]

[a] A. Padrayuttawat, T. Yoshizawa, H. Tamura, and T. Tokunaga 1997. "Optical Isomers and Odor Thresholds of Volatile Constituents in *Citrus sudachi*," Food Sci. Technol. Int. Tokyo,3(4):402–408

[b]Aurochemicals, "Safety Data Sheet (S)-Beta pinene," Material Safety Data Sheet, 2012. https://shrinkwrapcontainments.com/Images/media/SDS Shrink Film.pdf.

[c] J. Zhu, Y. Niu, and Z. Xiao 2022. "Aromatic Profiles and Enantiomeric Distributions of Chiral Volatile Compounds in Pu-Erh Tea," J. Agric. Food Chem. 70(27): 8395–8408

[d] Boelens, M. H., van Gemert, L. 1993. "Sensory Properties of Optical Isomers." Perfurmer Flavorist 18: 1–16

[e] Brenna, Elisabetta, Claudio Fuganti, and Stefano Serra. 2003. "Enantioselective Perception of Chiral Odorants." Tetrahedron Asymmetry 14(1): 1–42

[f] Koppenhoefer, B. et al. 1994. "Enantiomeric Odor Differences and Gas Chromatographic Properties of Flavors and Fragrances." Perfumer & flavorist 19(5): 1–14

Table S3: Percentage enantiomeric composition (EC%) of the reference sweet and bitter orange EOs obtained with optimized methods on the three investigated columns.

	REFERENCE ION (m/z)	BO_RIF			SO_RIF		
		DEX DET-Beta_25m	DEX DET-Beta_15m	DEX DET-Beta_10m	DEX DET-Beta_25m	DEX DET-Beta_15m	DEX DET-Beta_10m
**(1*R*)-(+)-β-Pinene**	93.0	3.5	3.9	3.8	60.6	58.2	57.2
**(1*S*)-(-)- β-Pinene**		96.5	96.1	96.2	39.4	41.8	42.8
**(1*R*,5*R*)-(+)-Sabinene**	91.0	10.7	10.6	12.1	96.52	96.1	96.1
**(1*S*,5*S*)-(-)-Sabinene**		89.3	89.4	87.9	3.48	3.9	3.9
**(*S*)-Limonene**	68.0	0.6	0.7	0.6	0.6	0.7	0.6
**(*R*)-Limonene**		99.4	99.3	99.4	99.4	99.3	99.4
**(*R*)-(-)-Linalool**	71.0	92.2	91.91	91.2	36.9	36.4	37.7
**(*S*)-(+)-Linalool**		7.8	8.09	8.8	63.1	63.6	62.2
**(*R*)-(-)-Linalyl acetate**	80.0	98.8	98.37	98.6	0.0	0.00	0.00
**(*S*)-(+)-Linalyl acetate**		1.2	1.63	1.4	0.0	0.00	0.00
**(*S*)-(-)-α-Terpineol**	59.0	20.4	22.6	23.2	0.9	0.1	0.1
**(*R*)-(+)- α-Terpineol**		79.6	77.5	76.9	99.1	99.9	99.9
**δ-3-carene (% TIC)**		0.02	0.03	0.03	0.08	0.1	0.09

Table S4: Percentage enantiomeric composition (EC%) and percentage relative composition (%TIC) ± standard deviation (n=3) of the target compounds for the reference commercial EOs samples. N.d: not detected. BO_RIF: reference of an authentic BO EO; SO_RIF: reference of an authentic SO EO.

SAMPLE	*(1R)-(+)-β-Pinene*	*(1S)-(-)- β-Pinene*	*(1R,5R)-(+)-Sabinene*	*(1S,5S)-(-)-Sabinene*	*(S)-Limonene*	*(R)-Limonene*	*(R)-(-)-Linalool*	*(S)-(+)-Linalool*	*(R)-(-)-Linalyl acetate*	*(S)-(+)-Linalyl acetate*	*(S)-(-)-α-Terpineol*	*(R)-(+)- α-Terpineol*	*δ-3-Carene (% TIC)*	*β--Pinene (%TIC)*	*Sabinene (%TIC)*	*Limonene (%TIC)*	*Linalool (%TIC)*	*Linalyl acetate (%TIC)*	*α-terpineol (%TIC)*
BO_RIF	3.48±0.09	96.53±0.09	10.73±0.21	89.27±0.21	0.63±0.21	99.38±0.01	92.23±0.01	7.77±0.03	98.84±0.03	1.16±0.01	20.4±0.01	79.6±0.17	0.02±0.01	0.32±0.01	0.23±0.01	90.14±0.06	0.43±0.02	0.78±0.01	0.17±0.01
BO_1	31.46±0.16	68.54±0.16	78.37±0.81	21.64±0.81	0.72±0.01	99.29±0.01	33.04±0.35	66.96±0.35	99.07±0.13	0.94±0.13	9.79±0.54	90.21±0.54	0.04±0.01	0.44±0.01	0.25±0.01	93.1±0.1	0.41±0.01	0.71±0.02	0.15±0.01
BO_2	2.312±0.17	97.68±0.17	62.45±0.44	37.55±0.44	0.63±0.01	99.38±0.01	55.5±1.13	44.5±1.13	96.71±0.20	3.29±0.20	1.65±0.42	98.35±0.42	n.d.	0.33±0.01	0.19±0.01	89.0±0.26	0.5±0.01	0.76±0.03	0.19±0.01
BO_3	4.87±0.24	95.13±0.24	36.75±0.64	63.25±0.64	0.67±0.01	99.34±0.01	74.86±0.08	25.14±0.08	99.07±0.37	0.94±0.37	14.63±0.98	85.38±0.98	0.02±0.01	0.28±0.01	0.11±0.01	87.43±0.23	0.13±0.01	0.49±0.01	0.14±0.01
BO_4	5.07±0.08	94.93±0.08	76.59±0.01	23.42±0.01	0.66±0.01	99.34±0.01	44.30±0.11	55.71±0.11	99.01±0.07	0.99±0.07	13.17±0.06	86.83±0.06	0.08±0.01	0.27±0.01	0.19±0.01	86.78±0.49	0.49±0.01	0.99±0.05	0.22±0.01
BO_5	4.36±0.07	95.64±0.07	81.88±0.25	18.13±0.25	0.65±0.01	99.36±0.01	48.87±0.02	51.14±0.02	99.53±0.14	0.47±0.14	13.63±1.04	86.38±1.04	0.12±0.01	0.64±0.01	0.31±0.01	85.22±0.04	0.68±0.01	1.53±0.01	0.26±0.01
BO_6	4.35±0.16	95.66±0.16	81.68±0.41	18.32±0.41	0.65±0.01	99.35±0.01	49.58±0.10	50.42±0.10	99.44±0.21	0.56±0.21	13.73±0.28	86.28±0.28	0.12±0.01	0.86±0.01	0.45±0.01	89.55±0.08	0.44±0.01	1.14±0.01	0.17±0.01
BO_7	4.94±0.02	95.07±0.02	61.49±2.06	38.52±2.06	0.67±0.01	99.34±0.01	46.79±0.09	53.22±0.09	98.86±0.22	1.15±0.22	13.43±0.13	86.58±0.13	0.02±0.01	0.94±0.01	0.47±0.01	88.72±0.04	0.49±0.01	1.23±0.01	0.2±0.01
BO_8	4.87±0.01	95.13±0.01	73.9±0.48	26.1±0.48	0.65±0.01	99.35±0.01	46.28±0.66	53.72±0.66	99.5±0.71	0.5±0.71	15.03±0.13	84.98±0.13	0.11±0.01	0.37±0.01	0.11±0.01	53.14±0.78	0.53±0.01	1.59±0.07	0.21±0.01
BO_9	5.91±0.09	94.10±0.09	71.31±1.27	28.70±1.27	0.67±0.01	99.34±0.01	43.29±0.03	56.71±0.03	98.43±0.20	1.57±0.20	8.07±1.23	91.93±1.23	0.04±0.01	0.98±0.02	0.32±0.01	89.9±0.06	0.5±0.01	1.07±0.01	0.15±0.01
BO_10	4.93±0.09	95.08±0.09	81.42±0.25	18.59±0.25	0.65±0.01	99.36±0.01	46.75±0.16	53.26±0.16	99.40±0.18	0.61±0.18	8.38±0.66	91.62±0.66	0.08±0.01	0.4±0.01	0.15±0.1	68.51±0.4	0.51±0.18	1.65±0.02	0.41±0.02
BO_11	4.93±0.24	95.07±0.24	81.64±0.23	18.37±0.23	0.64±0.01	99.37±0.01	46.51±0.03	53.49±0.03	99.81±0.27	0.19±0.27	9.08±0.18	90.93±0.18	0.08±0.01	0.63±0.01	0.21±0.01	93.22±0.18	0.39±0.01	0.87±0.02	0.18±0.01
BO_12	5.67±0.01	94.33±0.01	83.61±0.51	16.39±0.51	0.64±0.01	99.36±0.01	41.55±0.39	58.46±0.39	99.54±0.11	0.47±0.11	7.66±0.66	92.34±0.66	0.1±0.01	0.59±0.01	0.19±0.01	93.99±0.05	0.39±0.03	0.75±0.01	0.16±0.01
BO_13	5.78±0.01	94.23±0.01	84.15±0.93	15.86±0.93	0.64±0.01	99.37±0.01	42.14±0.86	57.86±0.86	99.61±0.26	0.40±0.26	7.64±0.69	92.36±0.69	0.1±0.01	0.6±0.01	0.24±0.01	92.74±0.04	0.45±0.01	0.77±0.04	0.21±0.01
BO_14	4.52±0.14	95.48±0.14	79.19±0.08	20.82±0.08	0.67±0.01	99.33±0.01	48.74±0.26	51.27±0.26	98.72±0.02	1.29±0.02	13.88±0.78	86.13±0.78	0.1±0.01	0.61±0.01	0.25±0.01	92.99±0.13	0.42±0.01	0.77±0.04	0.22±0.01
BO_15	5.56±0.40	94.45±0.40	37.23±0.14	62.77±0.14	0.69±0.01	99.32±0.01	75.10±0.01	24.91±0.01	98.48±0.11	1.52±0.11	16.19±0.13	83.82±0.13	0.02±0.01	0.79±0.01	0.37±0.01	83.18±0.5	0.67±0.01	1.78±0.07	0.28±0.01
BO_16	5.83±0.12	94.18±0.12	38.36±0.62	61.64±0.62	0.69±0.01	99.32±0.01	75.01±0.45	25.0±0.45	98.25±0.30	1.75±0.30	16.2±0.28	83.8±0.28	0.02±0.01	0.29±0.01	0.21±0.01	86.45±0.37	0.55±0.01	1.11±0.01	0.24±0.01
BO_17	4.25±0.11	95.76±0.11	80.6±0.49	19.4±0.49	0.67±0.01	99.33±0.01	48.86±0.01	51.14±0.01	98.85±0.08	1.16±0.08	14.32±0.64	85.69±0.64	0.11±0.01	0.31±0.01	0.21±0.01	86.49±0.22	0.55±0.01	1.14±0.01	0.27±0.01
BO_18	4.51±0.13	95.50±0.13	79.84±0.18	20.16±0.18	0.68±0.01	99.33±0.01	49.1±0.30	50.9±0.30	98.53±0.23	1.48±0.23	14.78±0.12	85.23±0.12	0.12±0.01	0.97±0.02	0.45±0.01	84.63±0.16	0.69±0.03	1.76±0.02	0.27±0.01
BO_19	4.42±0.01	95.58±0.01	80.4±0.40	19.6±0.40	0.67±0.01	99.33±0.01	49.16±0.07	50.84±0.07	99.1±0.13	0.9±0.13	14.33±0.47	85.68±0.47	0.12±0.01	1.04±0.01	0.47±0.01	84.65±0.3	0.66±0.01	1.74±0.04	0.3±0.04
BO_20	4.53±0.04	95.48±0.04	37.59±0.05	62.42±0.5	0.67±0.01	99.34±0.01	74.40±0.25	25.51±0.25	98.48±0.76	1.52±0.76	15.56±0.27	84.44±0.27	0.02±0.01	0.95±0.01	0.46±0.01	85.89±0.13	0.63±0.01	1.58±0.01	0.26±0.01
BO_21	3.33±0.06	96.68±0.06	9.43±0.27	90.57±0.27	0.62±0.01	99.39±0.01	92.73±0.49	7.28±0.49	99.16±0.01	0.84±0.01	19.09±0.23	80.92±0.23	0.01±0.01	0.91±0.01	0.25±0.01	92.06±0.13	0.49±0.04	1.15±0.01	0.07±0.01
BO_22	3.39±0.02	96.62±0.02	10.58±0.07	89.42±0.07	0.66±0.01	99.34±0.01	95.74±0.26	4.27±0.26	99.30±0.43	0.71±0.43	22.58±1.55	77.43±1.55	0.01±0.01	0.91±0.01	0.24±0.01	91.92±0.12	0.48±0.01	1.23±0.02	0.07±0.01
BO_23	3.35±0.03	96.65±0.03	11.09±0.54	88.91±0.54	0.67±0.01	99.33±0.01	95.65±0.05	4.36±0.05	98.91±0.24	1.09±0.24	22.73±0.78	77.27±0.78	0.01±0.01	0.94±0.01	0.23±0.01	92.62±0.01	0.47±0.01	1.15±0.03	0.07±0.01
BO_24	4.02±0.02	95.99±0.02	49.32±0.74	50.69±0.74	0.64±0.01	99.36±0.01	10.58±0.01	89.43±0.01	98.66±0.01	1.34±0.01	0.33±0.46	99.68±0.46	0.04±0.01	0.92±0.04	0.23±0.01	91.82±0.04	0.49±0.01	1.25±0.01	0.08±0.08
BO_25	3.24±0.07	96.76±0.07	8.27±0.06	91.73±0.06	0.63±0.01	99.37±0.01	97.23±0.42	2.77±0.42	98.7±0.17	1.3±0.17	20.49±0.88	79.52±0.88	n.d.	0.84±0.01	0.25±0.01	91.84±0.16	0.7±0.01	1.14±0.02	0.16±0.01
BO_27	3.49±0.10	96.51±0.10	8.21±0.32	91.80±0.32	0.62±0.01	99.39±0.01	96.96±0.09	3.05±0.09	97.56±0.37	2.45±0.37	41.38±0.64	58.63±0.64	n.d.	0.89±0.01	0.26±0.01	93.57±0.01	0.71±0.03	0.99±0.01	0.17±0.01

SAMPLE	**(1R)-(+)-β-Pinene**	**(1S)-(-)- β-Pinene**	**(1R,5R)-(+)-Sabinene**	**(1S,5S)-(-)-Sabinene**	**(S)-Limonene**	**(R)-Limonene**	**(R)-(-)-Linalool**	**(S)-(+)-Linalool**	**(R)-(-)-Linalyl acetate**	**(S)-(+)-Linalyl acetate**	**(S)-(-)-α-Terpineol**	**(R)-(+)- α-Terpineol**	**δ-3-Carene (% TIC)**	**β--Pinene (%TIC)**	**Sabinene (%TIC)**	**Limonene (%TIC)**	**Linalool (%TIC)**	**Linalyl acetate (%TIC)**	**α-terpineol (%TIC)**
BO_28	3.69±0.35	96.32±0.35	77.52±0.34	22.48±0.34	0.63±0.01	99.37±0.01	91.48±0.11	8.52±0.11	99.11±0.21	0.90±0.21	n.d.	n.d.	n.d.	0.9±0.01	0.27±0.01	93.84±0.04	0.64±0.01	0.91±0.01	0.08±0.01
BO_29	3.69±0.35	96.32±0.35	77.52±0.34	22.48±0.34	0.63±0.01	99.37±0.01	91.48±0.11	8.52±0.11	99.11±0.21	0.90±0.21	n.d.	n.d.	n.d.	0.87±0.01	0.23±0.01	95.05±0.1	0.24±0.01	1.14±0.03	0.0
SO_RIF	60.56±0.57	39.45±0.57	97.76±0.06	2.25±0.06	0.64±0.01	99.36±0.01	36.88±0.25	63.13±0.25	n.d.	n.d.	0.9±1.27	99.1±1.27	0.08±0.01	0.03±0.01	0.47±0.01	95.09±0.06	0.48±0.01	n.d.	0.02±0.01
SO_1	16.70±0.23	83.31±0.23	89.83±0.04	10.17±0.04	0.69±0.01	99.32±0.01	7.76±0.05	92.25±0.05	100±0.01	0±0.01	9.26±0.47	90.75±0.47	0.14±0.01	0.3±0.01	0.38±0.01	76.14±0.71	1.58±0.04	0.07±0.04	0.29±0.16
SO_2	23.43±0.25	76.57±0.25	87.82±0.47	12.19±0.47	0.69±0.01	99.32±0.01	6.58±0.06	93.43±0.06	99.9±0.01	0.1±0.01	8.74±0.48	91.26±0.48	0.14±0.01	0.2±0.01	0.29±0.01	60.58±1.96	2.14±0.03	0.1±0.01	0.48±0.03
SO_3	55.8±0.96	44.2±0.96	98.24±0.57	1.77±0.57	0.62±0.01	99.39±0.01	8.31±0.30	91.70±0.30	n.d.	n.d.	0.64±0.90	99.37±0.90	0.18±0.01	0.03±0.01	0.36±0.01	95.61±0.11	0.33±0.01	n.d.	0.03±0.01
SO_4	49.45±1.07	50.55±1.07	96.1±0.07	3.9±0.07	0.64±0.01	99.36±0.01	9.09±0.31	90.91±0.31	99.99±0.01	0.001±0.01	0.001±0.01	99.99±0.01	0.14±0.01	0.04±0.01	0.37±0.01	93.78±0.06	0.47±0.01	0.02±0.01	0.04±0.04
SO_5	19.22±0.44	80.78±0.44	85.55±0.39	14.46±0.39	0.66±0.01	99.34±0.01	12.02±0.21	87.98±0.21	99.99±0.01	0.001±0.01	1.49±0.1	98.51±0.1	0.1±0.01	0.09±0.01	0.28±0.01	88.42±0.24	0.72±0.01	0.07±0.01	0.11±0.01
SO_6	25.73±0.1	74.27±0.1	88.98±0.49	11.03±0.49	0.65±0.01	99.35±0.01	10.89±0.43	89.115±0.43	99.99±0.01	0.001±0.01	3.27±1.24	96.74±1.24	0.14±0.01	0.1±0.01	0.36±0.01	89.99±0.06	0.61±0.02	0.06±0.01	0.07±0.07
SO_7	18.99±0.21	81.02±0.21	86.17±0.49	13.83±0.49	0.66±0.01	99.34±0.01	11.02±0.08	88.99±0.08	99.99±0.01	0.001±0.01	5.62±1.11	94.39±1.11	0.11±0.01	0.11±0.01	0.3±0.01	88.51±0.13	0.75±0.01	0.07±0.01	0.09±0.01
SO_8	22.23±0.1	77.77±0.1	89.01±0.37	11.0±0.37	0.65±0.01	99.35±0.01	55.24±0.1	44.76±0.1	99.99±0.01	0.001±0.01	2.67±1.20	97.34±1.20	0.14±0.01	0.13±0.01	0.39±0.01	89.03±0.08	1.04±0.06	0.06±0.01	0.03±0.01
SO_9	20.55±0.81	79.45±0.81	85.7±0.47	14.3±0.47	0.67±0.01	99.34±0.01	55.23±0.2	44.77±0.2	99.99±0.01	0.001±0.01	11.24±0.07	88.76±0.07	0.12±0.01	0.13±0.01	0.35±0.01	81.62±0.54	1.27±0.01	0.07±0.01	0.05±0.01
SO_10	31.59±0.23	68.41±0.23	94.82±0.55	5.18±0.55	0.64±0.01	99.37±0.01	47.22±0.25	52.79±0.25	99.99±0.01	0.001±0.01	21.59±0.43	78.42±0.43	0.17±0.01	0.07±0.01	0.28±0.01	93.88±0.2	0.65±0.01	0.02±0.01	0.15±0.02
SO_11	30.25±0.29	69.76±0.29	94±0.23	6±0.23	0.6±0.01	99.36±0.01	47.23±0.12	52.78±0.12	99.99±0.01	0.001±0.01	20.88±0.1	79.12±0.1	0.16±0.01	0.07±0.01	0.27±0.01	92.74±0.05	0.72±0.05	0.02±0.01	0.17±0.01
SO_12	52.8±1.9	47.2±1,9	97.660.3	2.34±0.3	0.63±0.01	99.38±0.01	6.87±0.01	93.13±0.01	n.d.	n.d.	0±0.01	100±0.01	0.12±0.01	0.04±0.01	0.32±0.01	95.04±0.06	0.53±0.01	n.d.	0.05±0.01
SO_13	52.21±0.78	47.79±0.78	97.33±0.15	2.68±0.15	0.62±0.01	99.38±0.01	6.87±0.07	93.13±0.07	n.d.	n.d.	5.29±1.44	94.71±1.44	0.12±0.01	0.04±0.01	0.32±0.01	95.21±0.08	0.49±0.01	n.d.	0.05±0.03
SO_14	56.39±0.47	43.61±0.47	97.22±0.3	2.78±0.3	0.62±0.01	99.38±0.01	6.53±0.14	93.47±0.14	n.d.	n.d.	4.82±1.46	95.18±1.46	0.14±0.01	0.03±0.01	0.4±0.01	93.49±0.01	0.7±0.09	n.d.	0.08±0.01
SO_15	61.08±0.66	38.92±0.66	97.39±0.66	2.61±0.66	0.64±0.01	99.36±0.01	36.85±0.58	63.15±0.58	n.d.	n.d.	0.001±0.01	99.99±0.01	0.08±0.01	0.03±0.01	0.49±0.01	95.18±0.01	0.54±0.04	n.d.	0.02±0.01
SO_16	75.01±3.13	24.99±3.13	97.52±0.87	2.49±0.87	0.63±0.01	99.37±0.01	5.79±0.64	94.22±0.64	n.d.	n.d.	0.001±0.01	99.99±0.01	0.09±0.01	0.02±0.01	0.28±0.01	92.14±0.16	0.45±0.03	n.d.	0.04±0.01
SO_17	80.15±0.03	19.85±0.03	96.88±0.15	3.13±0.15	0.63±0.01	99.37±0.01	6.18±0.31	93.82±0.31	n.d.	n.d.	0.57±0.8	99.44±0.8	0.09±0.01	0.01±0.01	0.27±0.01	90.04±0.08	0.51±0.06	n.d.	0.05±0.01
SO_18	31.9±0.14	68.1±0.14	96.75±0.88	3.25±0.88	0.63±0.01	99.37±0.01	81.6±0.03	18.4±0.03	n.d.	n.d.	24.61±0.1	75.39±0.1	0.12±0.01	0.03±0.01	0.23±0.01	95.31±0.06	0.78±0.01	n.d.	0.19±0.01
SO_19	54.48±2.41	45.53±2.41	96.97±0.1	3.03±0.1	0.62±0.01	99.38±0.01	5.90±0.23	94.11±0.23	n.d.	n.d.	4.03±0.15	95.98±0.15	0.14±0.01	0.04±0.01	0.43±0.02	93.8±0.04	0.71±0.1	n.d.	0.08±0.01

Table S5: Summary of the conventional and optimized GC analyses conditions adopted in this study.

PARAMETERS	DEX DET-Beta_25m conventional method	DEX DET-Beta_25m optimized method	DEX DET-Beta_15m	DEX DET-Beta_10m
Program temperature	50.0 °C/2 °C min^{-1}/ 220 °C (2 min)	50.0 °C/3 °C min^{-1}/ 120 °C/10 °C min^{-1}/220 °C	50.0 °C/4 °C min^{-1}/ 120 °C/18 °C min^{-1}/220 °C	50.0 °C/5.48 °C min^{-1}/ 120 °C/25 °C min^{-1}/220 °C
Flow rate (mL min^{-1})	1	1	0.72	0.4
Split ratio	20:1	20:1	50:1	100:1
Analysis time (min)	87	31	22	16

Analytical Greenness report sheet

21/06/2023 16:02:48

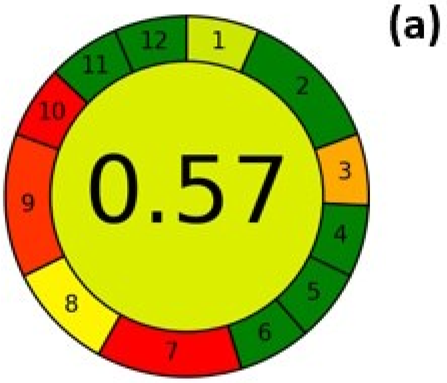

Criteria	Score	Weight
1. Direct analytical techniques should be applied to avoid sample treatment.	0.6	2
2. Minimal sample size and minimal number of samples are goals.	1.0	4
3. If possible, measurements should be performed in situ.	0.33	2
4. Integration of analytical processes and operations saves energy and reduces the use of reagents.	1.0	2
5. Automated and miniaturized methods should be selected.	1.0	2
6. Derivatization should be avoided.	1.0	2
7. Generation of a large volume of analytical waste should be avoided, and proper management of analytical waste should be provided.	0	4
8. Multi-analyte or multi-parameter methods are preferred versus methods using one analyte at a time.	0.48	3
9. The use of energy should be minimized.	0.1	4
10. Reagents obtained from renewable sources should be preferred.	0.0	2
11. Toxic reagents should be eliminated or replaced.	1.0	2
12. Operator's safety should be increased.	1.0	2

Figure S1: Pictograms and reports obtained with AGREE for the conventional method on the 25 m column.

(b)

Analytical Greenness report sheet

21/06/2023 15:58:35

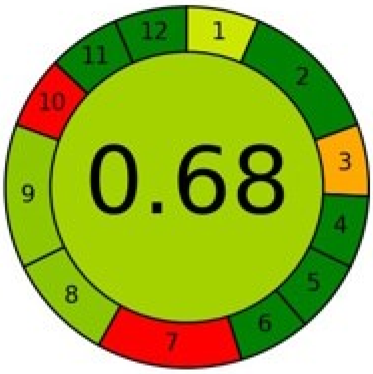

Criteria	Score	Weight
1. Direct analytical techniques should be applied to avoid sample treatment.	0.6	2
2. Minimal sample size and minimal number of samples are goals.	1.0	4
3. If possible, measurements should be performed in situ.	0.33	2
4. Integration of analytical processes and operations saves energy and reduces the use of reagents.	1.0	2
5. Automated and miniaturized methods should be selected.	1.0	2
6. Derivatization should be avoided.	1.0	2
7. Generation of a large volume of analytical waste should be avoided, and proper management of analytical waste should be provided.	0	4
8. Multi-analyte or multi-parameter methods are preferred versus methods using one analyte at a time.	0.73	3
9. The use of energy should be minimized.	0.72	4
10. Reagents obtained from renewable sources should be preferred.	0.0	2
11. Toxic reagents should be eliminated or replaced.	1.0	2
12. Operator's safety should be increased.	1.0	2

Figure S2: Pictograms and reports obtained with AGREE and for the optimized method on the 25 m column.

(c)

Analytical Greenness report sheet

21/06/2023 16:09:10

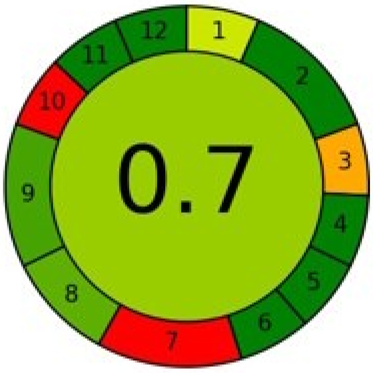

Criteria	Score	Weight
1. Direct analytical techniques should be applied to avoid sample treatment.	0.6	2
2. Minimal sample size and minimal number of samples are goals.	1.0	4
3. If possible, measurements should be performed in situ.	0.33	2
4. Integration of analytical processes and operations saves energy and reduces the use of reagents.	1.0	2
5. Automated and miniaturized methods should be selected.	1.0	2
6. Derivatization should be avoided.	1.0	2
7. Generation of a large volume of analytical waste should be avoided, and proper management of analytical waste should be provided.	0	4
8. Multi-analyte or multi-parameter methods are preferred versus methods using one analyte at a time.	0.82	3
9. The use of energy should be minimized.	0.87	4
10. Reagents obtained from renewable sources should be preferred.	0.0	2
11. Toxic reagents should be eliminated or replaced.	1.0	2
12. Operator's safety should be increased.	1.0	2

Figure S3: Pictograms and reports obtained with AGREE and for the optimized method on the 15 m column.

Analytical Greenness report sheet

21/06/2023 16:12:55

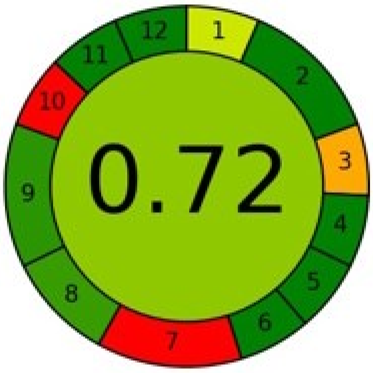

(d)

Criteria	Score	Weight
1. Direct analytical techniques should be applied to avoid sample treatment.	0.6	2
2. Minimal sample size and minimal number of samples are goals.	1.0	4
3. If possible, measurements should be performed in situ.	0.33	2
4. Integration of analytical processes and operations saves energy and reduces the use of reagents.	1.0	2
5. Automated and miniaturized methods should be selected.	1.0	2
6. Derivatization should be avoided.	1.0	2
7. Generation of a large volume of analytical waste should be avoided, and proper management of analytical waste should be provided.	0	4
8. Multi-analyte or multi-parameter methods are preferred versus methods using one analyte at a time.	0.89	3
9. The use of energy should be minimized.	0.92	4
10. Reagents obtained from renewable sources should be preferred.	0.0	2
11. Toxic reagents should be eliminated or replaced.	1.0	2
12. Operator's safety should be increased.	1.0	2

Figure S4: Pictograms and reports obtained with AGREE and for the optimized method on the 10 m column.
